# Pericyte Loss Leads to Capillary Stalling Through Increased Leukocyte-Endothelial Cell Interaction in the Brain

**DOI:** 10.3389/fncel.2022.848764

**Published:** 2022-03-11

**Authors:** Young-Geun Choe, Jin-Hui Yoon, Jongyoon Joo, Bokyung Kim, Seon Pyo Hong, Gou Young Koh, Dong-Seok Lee, Wang-Yuhl Oh, Yong Jeong

**Affiliations:** ^1^Department of Bio and Brain Engineering, Korea Advanced Institute of Science and Technology, Daejeon, South Korea; ^2^KI for Health Science and Technology, Korea Advanced Institute of Science and Technology, Daejeon, South Korea; ^3^Center for Vascular Research, Institute for Basic Science, Daejeon, South Korea; ^4^Department of Mechanical Engineering, Korea Advanced Institute of Science and Technology, Daejeon, South Korea; ^5^School of Life Sciences and Biotechnology, College of Natural Sciences, Kyungpook National University, Daegu, South Korea; ^6^BK21 FOUR KNU Creative BioResearch Group, School of Life Sciences, Kyungpook National University, Daegu, South Korea; ^7^Graduate School of Medical Science and Engineering, Korea Advanced Institute of Science and Technology, Daejeon, South Korea

**Keywords:** capillary stalling, pericyte, leukocyte-endothelial cell interaction, cerebral endothelial glycocalyx, leukocyte adhesion molecules

## Abstract

The neurovascular unit is a functional unit composed of neurons, glial cells, pericytes, and endothelial cells which sustain brain activity. While pericyte is a key component of the neurovascular unit, its role in cerebral blood flow regulation remains elusive. Recently, capillary stalling, which means the transient interruption of microcirculation in capillaries, has been shown to have an outsized impact on microcirculatory changes in several neurological diseases. In this study, we investigated capillary stalling and its possible causes, such as the cerebral endothelial glycocalyx and leukocyte adhesion molecules after depleting pericytes postnatally in mice. Moreover, we investigated hypoxia and gliosis as consequences of capillary stalling. Although there were no differences in the capillary structure and RBC flow, longitudinal optical coherence tomography angiography showed an increased number of stalled segments in capillaries after pericyte loss. Furthermore, the extent of the cerebral endothelial glycocalyx was decreased with increased expression of leukocyte adhesion molecules, suggesting enhanced interaction between leukocytes and endothelial cells. Finally, pericyte loss induced cerebral hypoxia and gliosis. Cumulatively, the results suggest that pericyte loss induces capillary stalling through increased interaction between leukocytes and endothelial cells in the brain.

## Introduction

A stable and adequate blood supply through the capillaries is crucial to meet the high energy demands of the brain (Dawson, 1999; Fehm et al., 2006; Berthiaume et al., 2018b). The neurovascular unit is composed of neurons, glial cells, pericytes, and endothelial cells that interact together to properly sustain brain activity through cerebral microcirculation (Iadecola, 2017). Previous studies have shown that pericyte loss decreases cerebral blood flow (CBF) and blood flow responses, which suggest the contribution of pericyte to cerebral microcirculation (Bell et al., 2010; Hall et al., 2014; Mishra et al., 2016; Sweeney et al., 2016; Kisler et al., 2017; Nikolakopoulou et al., 2019). Furthermore, pericyte loss has been reported in various brain diseases, such as Alzheimer’s disease, stroke, vascular dementia, and traumatic brain injury (Dore-Duffy et al., 2000; Fernández-Klett et al., 2012; Sengillo et al., 2013; Hall et al., 2014; Miners et al., 2017; Ding et al., 2020). Although previous studies have suggested the involvement of pericyte in CBF maintenance and regulation, the mechanism by which pericyte affects CBF remains elusive.

Cerebral microcirculation is not homogeneous (Villringer et al., 1994; Kleinfeld et al., 1998), and transient interruption of blood flow called capillary stalling occurs in the capillaries (Erdener et al., 2017; Reeson et al., 2018). Recently, capillary stalling has been reported as a cause of CBF downregulation, and leukocyte plugging contributes to capillary stalling. Leukocyte plugging is induced by the increased adherence of leukocytes to endothelial cells, probably due to the increased interaction between leukocytes and leukocyte adhesion molecules (Cruz Hernández et al., 2019; El Amki et al., 2020). In particular, leukocyte adhesion molecules, such as vascular cell adhesion molecule 1 (VCAM1) and intercellular adhesion molecule 1 (ICAM1), which mediate the arrest of rolling leukocytes in the blood, are known to be increasingly expressed under inflammatory condition, leading to leukocyte adhesion (Shapiro et al., 2010; Takeshita and Ransohoff, 2012).

Recently, it was shown that the extent of the cerebral endothelial glycocalyx was related to capillary stalling (Yoon et al., 2022). The endothelial glycocalyx is a scaffolding network of proteoglycans, glycoproteins, glycosaminoglycans, and associated plasma proteins on the luminal side of the vascular endothelium (Weinbaum et al., 2007). The endothelial glycocalyx serves as a barrier that limits the interaction between leukocytes and leukocyte adhesion molecules expressed on the endothelial cell surface (Reitsma et al., 2007; Uchimido et al., 2019).

In this study, we aimed to investigate the effect of pericyte loss on capillary stalling and its possible causes, such as the cerebral endothelial glycocalyx and leukocyte adhesion molecules after depleting pericytes postnatally. We also investigated hypoxia and gliosis as consequences of capillary stalling.

## Materials and Methods

### Animal Preparation and Experimental Procedures

The animal care and experimental procedures in this study were performed with the approval of the Institutional Animal Care and Use Committee (No. KA2018-32, KA2021-46) of KAIST, and in accordance with the ARRIVE guidelines (Percie du Sert et al., 2020).

PDGFRβ-CreER^T2^ mice (Sheikh et al., 2015) and ROSA26-DTA mice (Sheikh et al., 2015) were transferred, maintained in a specific pathogen-free facility of KAIST Laboratory Animal Resource Center, and used after more than 10 generations of back-crossing into C57BL/J background. PDGFRβ-CreER^T2^ mice and ROSA26-DTA mice were crossed to deplete pericytes by diphtheria toxin A (DTA) expression in a PDGFRβ-dependent tamoxifen-inducible manner (DTA^*i*△PC^) (Park et al., 2017). Both CreER^T2^- and loxP/loxP-positive, vehicle (corn oil) administered littermates were defined as control mice in each experiment. Male and female mice were used indiscriminately in the experiments. To deplete pericytes, 2 mg of tamoxifen (Sigma, T5648) dissolved in corn oil (Sigma, C8267) was injected intraperitoneally in 8-week-old DTA^*i*△PC^ mice for five consecutive days. After 2 weeks, the brain was collected to perform immunohistochemistry, RNA-sequencing (RNA-Seq), and western blotting after imaging.

### Cranial Window Surgery

To implant the chronic cranial window for *in vivo* imaging, the mice were anesthetized with the mixture of Zoletil (30 mg/kg) and xylazine (10 mg/kg) *via* intraperitoneal injection and a cover glass and head plate were implanted on the right primary somatosensory cortex at 6 weeks of age, as previously described (Goldey et al., 2014). Body temperature was maintained at 36.5–37.5°C throughout the experiment using a heating pad and a rectal feedback probe (Physitemp Instruments, TCAT-2LV). Imaging was performed after 2 weeks of recovery.

### *Ex vivo* Measurement of Capillary Stalling

DiI-coated 4-μm microspheres, which label stalled segments in capillaries (Nuytemans et al., 2018), were intravenously injected under isoflurane anesthesia, 3 h before the sacrifice. Specifically, we coated 4-μm microspheres (Thermo Fisher Scientific, F8858) with DiI solution (Thermo Fisher Scientific, D282) as previously described (Reeson et al., 2018). After evaporation and reconstitution in 0.9% saline, 40 μL of DiI-coated microspheres were intravenously injected under isoflurane anesthesia (5% for induction, and 1.5–2% for maintenance with a gas mixture of 40% oxygen and 60% air). After 3 h, the mice were deeply anesthetized with the mixture of Zoletil (30 mg/kg) and xylazine (10 mg/kg) and intravenously perfused with 100 μL of DyLight 488 tomato lectin (Vector Labs, DL-1174) for 5 min to label vascular structures (Robertson et al., 2015). Next, we performed transcardial perfusion with ice-cold phosphate-buffered saline (PBS) followed by 4% paraformaldehyde (PFA) in PBS. The extracted brain was post-fixed with 4% PFA for 12 h at 4°C and transferred to a 30% (w/v) sucrose solution for cryoprotection. The samples were coronally-sliced into 30 μm thick sections. Brain slices were mounted using Vectashield mounting medium (Vector Labs, H-1000). A confocal microscopy system (Nikon, A1HD25) with a Plan Apo 20 × /0.75 objective lens and a field of view of 634.88 × 634.88 μm, and a slide scanner (Zeiss, AxioScan Z1) with a Plan Apo 10 × /0.45 objective lens were utilized for imaging brain slices. Imaging and analysis were performed in the primary somatosensory cortex according to the mouse brain atlas (Paxinos and Franklin, 2001), and the measurements from at least 10 slides were averaged for each mouse.

### *In vivo* Measurement of Capillary Stalling

A swept-source optical coherence tomography (OCT) system with a center wavelength of 1.3 μm and an A-scan rate of 240 kHz, was used (Shin et al., 2018). The field of view of optical coherence tomography angiography (OCTA) imaging was 1 × 1 mm. The focus of the OCT beam was positioned approximately 220 μm below the cortical surface where the capillaries were embedded. Serial OCT en-face angiograms were continuously acquired every 6.3 s for 63 s to investigate temporal changes in microcirculation. Each angiogram consisted of 800 A-scans (in the x-direction) and 250 B-scans (in the y-direction). The axial and lateral resolutions of the system were 10 μm each. The inter B-scan time interval was determined to be 4.2 ms. Throughout the OCTA imaging experiment, the mice were anesthetized with isoflurane (5% for induction, and about 1.5% for maintenance with a gas mixture of 40% oxygen and 60% air), and physiological parameters, such as body temperature, heart rate, and oxygen saturation (SpO2) were monitored (Kent Scientific, PhysioSuite). Body temperature was maintained at 36.5–37.5°C with the use of a heating pad and a rectal feedback probe (Harvard Apparatus, 55-7020). Our procedures for OCTA data analysis have been described previously (Yoon et al., 2022). Maximum intensity projection of 3D OCT angiograms was performed in a depth direction over 150–300 μm below the cortical surface. A time-averaged OCT angiogram was generated by averaging all angiograms acquired within 63 s. The averaged angiograms were segmented using the Trainable Weka Segmentation plugin in Fiji software (Schindelin et al., 2012; Arganda-Carreras et al., 2017). The shadow of large pial vessels from the upper depth interval was removed from the image, and a binary mask of capillaries was generated. Capillary segments that showed a sudden drop of the signal at least once in the temporal series of the OCT angiogram were defined as stalled segments. Stalled segments were automatically identified using MATLAB (MathWorks), and confirmed by manual inspection. Capillary segments that were stalled both at baseline and after 2 weeks were defined as re-stalled segments.

### Measurement of Capillary Diameter, RBC Velocity, and RBC Volume Flux

After 2 weeks of recovery, *in vivo* imaging was performed before tamoxifen administration (baseline) and 2 weeks after the initial tamoxifen administration (after 2 weeks). A two-photon microscopy (TPM) system (Zeiss, LSM 510) with a femtosecond-pulsed tunable Ti:Sapphire laser (Coherent, Chameleon Ultra) and a Plan Apo 20 × /1.0 water immersion objective lens was used for imaging. Throughout the TPM imaging experiment, mice were anesthetized with isoflurane (5% for induction, and about 1.5% for maintenance with a gas mixture of 40% oxygen and 60% air), and physiological parameters, such as heart rate and SpO_2_ were monitored (Kent Scientific, PhysioSuite). Body temperature was maintained at 36.5–37.5°C with the use of a heating pad and a rectal feedback probe (Physitemp Instruments, TCAT-2LV). To image vessels, 70-kDa Texas Red dextran (Thermo Fisher Scientific, D1830; 1% (w/v) in saline) was intravenously injected at a volume of 100 μL to label blood plasma immediately before imaging. Imaging was performed at 150–300 μm below the cortical surface with a center wavelength of 900 nm (with a laser power of less than 50 mW). The measurements from at least ten capillaries were averaged for each mouse. For capillary diameter measurements, 5 diameter line scan images (scan time: 6.14 ms / line) were analyzed with Gaussian smoothing (σ = 3) and a Huang threshold method using the Fiji software (Kisler et al., 2017, 2018). For RBC velocity measurements, 5,000 velocity line scan images (scan time: 1.93 ms/line) were analyzed using the MATLAB Line-Scanning Particle Image Velocimetry (LS-PIV) algorithm which determines RBC displacements between pairs of line scan images using spatial cross-correlation analysis (Kim et al., 2012). For our image settings, the LS-PIV parameters used were windowsize = 518, number of averages (numavgs) = 200, skip amount (skipamt) = 25, and shift amount (shiftamt) = 1, as previously described (Kisler et al., 2018). For RBC volume flux measurements, capillary diameter and RBC velocity collected from a single capillary were used to define RBC volume flux $\vec{F}$ using the formula,


$$\vec{F}=\frac{\pi }{8}\,\vec{v}\,\left(0\right)\,d^{2}$$

where $\vec{v}\,\left(0\right)$ is RBC velocity, and *d* is capillary diameter, with the assumption that the flow in the capillary is laminar (Shih et al., 2009, 2012). The measurements from at least 10 capillaries were averaged for each mouse.

### Measurement of the Extent of the Cerebral Endothelial Glycocalyx

To label and image the cerebral endothelial glycocalyx, FITC-conjugated wheat germ agglutinin (WGA) lectin (Sigma, L4895; 0.1% (w/v) in saline) was intravenously injected 45 min before imaging at a dose of 6.25 mg/kg. Subsequently, 70-kDa Texas Red dextran was intravenously injected at a volume of 100 μL to label blood plasma immediately before imaging. Imaging was performed at 150–250 μm below the cortical surface with a center wavelength of 800 nm (with a laser power of less than 50 mW). A TPM system (Zeiss, LSM 510) with a femtosecond-pulsed tunable Ti:Sapphire laser (Coherent, Chameleon Ultra) and a Plan Apo 20 × /1.0 water immersion objective lens was used for imaging. Throughout the TPM imaging experiment, mice were anesthetized with isoflurane (5% for induction, and about 1.5% for maintenance with a gas mixture of 40% oxygen and 60% air), and physiological parameters, such as heart rate and SpO2 were monitored (Kent Scientific, PhysioSuite). Body temperature was maintained at 36.5–37.5°C with the use of a heating pad and a rectal feedback probe (Physitemp Instruments, TCAT-2LV).

To measure the extent of the cerebral endothelial glycocalyx, the intensity profiles of WGA lectin and dextran, which were vertical to the capillary wall, were averaged along the capillary wall using the Fiji software. The extent of the cerebral endothelial glycocalyx was defined as the area under the curve (AUC) of WGA lectin signal intensity between boundaries, as shown in Supplementary Figure 1 (Yoon et al., 2017). The measurements from at least ten capillaries were averaged for each mouse.

### Immunohistochemistry

All immunohistochemical analyses were performed in the primary somatosensory cortex, 2 weeks after the initial tamoxifen administration. The mice were deeply anesthetized with a mixture of Zoletil (30 mg/kg) and xylazine (10 mg/kg), and intravenously perfused with 100 μL of DyLight 488 tomato lectin (Vector Labs, DL-1174) for 5 min to label vascular structures. Next, we performed transcardial perfusion with ice-cold PBS followed by 4% PFA in PBS. The extracted brain was post-fixed with 4% PFA for 12 h at 4°C and transferred to a 30% (w/v)-sucrose solution for cryoprotection. The samples were sliced coronally at a thickness of 30 μm. Brain slices were blocked with 5% donkey (or goat) serum dissolved in PBST (0.3% Triton X-100 in PBS) at room temperature for 1 h and then incubated with the following primary antibodies at 4°C for 12 h: anti-CD13 (1:200; R&D Systems, AF2335), GLUT1 (1:200; Abcam, ab14683), GFAP (1:1,000; Abcam, ab4674), Tie2 (1:200; R&D Systems, AF762), for 24 h: VCAM1 (1:100; Sigma, CBL1300), and ICAM1 (1:100; Abcam, ab119871). After several washes with PBS, brain slices were incubated with the following secondary antibodies at 4°C for 12 h: anti-goat IgG, anti-rabbit IgG, anti-rat IgG (1:1,000; Jackson ImmunoResearch), and anti-chicken IgG (1:1,000; Abcam). To detect cerebral hypoxia, Hypoxyprobe-1 (Hypoxyprobe, HP7) was injected intraperitoneally 1 h before sacrificing the mice. To quantify the blood-brain barrier (BBB) leakage, Alexa Fluor 405 Cadaverine (Thermo Fisher Scientific, A30675) was intravenously injected under isoflurane anesthesia (5% for induction, and 1.5–2% for maintenance with a gas mixture of 40% oxygen and 60% air). After 3 h, the mice were sacrificed and brain tissue was harvested with PBS and PFA perfusion. Brain slices were incubated with anti-mouse IgG (1:1,000; Abcam) antibody at 4°C for 12 h. Brain slices were mounted using Vectashield mounting medium (Vector Labs, H-1000). Confocal microscopy (Nikon, A1HD25) with a Plan Apo 20 × /0.75 objective lens, a field of view of 317.44 × 317.44 μm (for CD13 and GFAP) or 634.88 × 634.88 μm (for GLUT1, Tie2, VCAM1, ICAM1, Hypoxyprobe-1, Cadaverine, and IgG), was utilized for imaging brain slices. Images were analyzed using the Fiji software. For analysis, lectin^+^-vessel mask was segmented using the Trainable Weka Segmentation plugin in Fiji software (Schindelin et al., 2012; Arganda-Carreras et al., 2017). Pericyte coverage was defined by the ratio of CD13^+^-pericyte area to lectin^+^-vessel masked area in the image. Capillary density was defined by the percentage of lectin^+^-vessel masked area in the image. Capillary length was defined by the length of skeletonized lectin^+^-vessel mask in the image, and adjusted as a scale of mm/mm^3^. To measure capillary tortuosity, lectin^+^-vessel mask was utilized along with Rapid Editable Analysis of Vessel Elements Routine (REAVER),^1^ an open-source tool to analyze vascular network, as previously described (Corliss et al., 2020). The measurements from at least 10 slides were averaged for each mouse.

### RNA-Seq of Brain Capillaries

We performed transcardial perfusion with ice-cold DEPC-treated water under deep anesthesia with the mixture of Zoletil (30 mg/kg) and xylazine (10 mg/kg). Capillaries were isolated from the extracted brain, as previously described (Ogata et al., 2020). Total RNA was isolated using easy-BLUE reagent (iNtRON Biotechnology, 17061). RNA quality was assessed by Agilent 2100 bioanalyzer using the RNA 6000 Nano Chip (Agilent Technologies), and RNA quantification was performed using ND-2000 Spectrophotometer (Thermo Fisher Scientific). The construction of library was performed using QuantSeq 3′ mRNA-Seq Library Prep Kit (Lexogen) according to the manufacturer’s instructions. In brief, each 500 ng total RNA were prepared and an oligo-dT primer containing an Illumina-compatible sequence at its 5′ end was hybridized to the RNA and reverse transcription was performed. After degradation of the RNA template, second strand synthesis was initiated by a random primer containing an Illumina-compatible linker sequence at its 5′ end. The double-stranded library was purified by using magnetic beads to remove all reaction components. The library was amplified to add the complete adapter sequences required for cluster generation. The finished library is purified from PCR components. High-throughput sequencing was performed as single-end 75 sequencing using NextSeq 500 (Illumina). QuantSeq 3′ mRNA-Seq reads were aligned using Bowtie2 (Langmead and Salzberg, 2012). Bowtie2 indices were either generated from genome assembly sequence or the representative transcript sequences for aligning to the genome and transcriptome. The alignment file was used for assembling transcripts, estimating their abundances and detecting differential expression of genes. Differentially expressed genes (DEGs) were determined based on counts from unique and multiple alignments using coverage in Bedtools (Quinlan and Hall, 2010). The read count data were processed based on quantile normalization method using EdgeR within a R software using Bioconductor (Gentleman et al., 2004). Significant DEGs were defined as *P* < 0.05. Gene classification was based on searches done by DAVID and Medline databases (Huang et al., 2009a,b). Gene set enrichment analysis (GSEA) was performed using GSEA software^2^ to find enriched gene sets. Enrichment analysis was performed by using gene set collections of the molecular signature database (MSigDB v7.4) obtained from the Broad Institute.^3^

### Western Blotting

After transcardial perfusion with ice-cold PBS under deep anesthesia with the mixture of Zoletil (30 mg/kg) and xylazine (10 mg/kg), protein lysates were extracted from the brain using an ice-cold PRO-PREP protein extraction solution (iNtRON Biotechnology, 17081) with protease/phosphatase inhibitor and EDTA (Thermo Fisher Scientific, 1861284). Protein quantification was performed using the Bradford assay (Bio-Rad), and the lysates containing 10–20 mg of protein were separated on 8–15% sodium dodecyl sulfate-polyacrylamide gel electrophoresis (SDS-PAGE), followed by transferring onto nitrocellulose membranes (Pall Corporation, 66485). After blocking with 5% skim milk (BD Biosciences, 232100), the membranes were incubated with following primary antibodies overnight at 4°C: anti-VCAM1 (1:2,000; Santa Cruz Biotechnology, sc-13160), ICAM1 (1:2,000; R&D Systems, AF796), Tie2 (1:2,000; R&D Systems, AF762), p-Tie2 (1:1,000; R&D Systems, AF2720), NFκB (1:1,000; Santa Cruz Biotechnology, sc-8008), p-NFκB (1:1,000; Cell Signaling Technology, 3039), Angiopoietin1 (1:2,000; R&D Systems, AF923), Angiopoietin2 (1:2,000; R&D Systems, AF623), Tie1 (1:2,000; R&D Systems, AF619), glyceraldehyde 3-phosphate dehydrogenase (GAPDH; 1:5,000; Santa Cruz Biotechnology, sc-25778). Then, the membranes were incubated with horseradish peroxidase-conjugated anti-rat, anti-rabbit, and anti-mouse antibodies (1:5,000, Thermo Fisher Scientific) at 4°C. After removing the secondary antibodies and washing with TBST (tris-buffered saline with 0.1% tween 20) for a few times, the band intensities were detected using the Clarity Western ECL Substrate (Bio-Rad, 1705061). Images were analyzed in an ImageJ software (NIH).

### Statistical Analyses

Statistical analyses were performed using GraphPad Prism 9 (GraphPad Software). Data are presented as mean ± standard deviation (SD) or median with interquartile range. Data were tested for normality using Shapiro-Wilk test. Differences between groups were tested using parametric or non-parametric statistics depending on the distribution of the data. For comparing two groups, Student’s *t*-test, Welch’s *t*-test or Mann-Whitney test were used. Statistical significance was set to *P* < 0.05. All statistical details are included in figure legends and Supplementary Table 1.

## Results

### Pericytes Are Efficiently Ablated in the Brain of DTA^*i*△PC^ Mice

**FIGURE 1:**
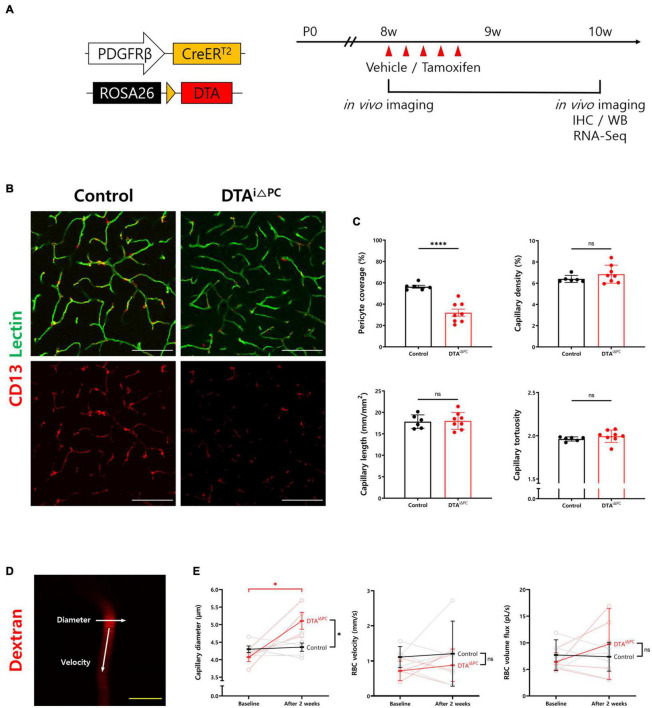
Pericyte coverage is reduced in the brain of DTA^*i*△PC^ mice. **(A)** Schematic diagram of the mouse model and experimental timeline for selective depletion of pericytes in brain capillaries by tamoxifen administration. Tamoxifen (2 mg) was treated intraperitoneally for five consecutive days in 8-week-old DTA^*i*△PC^ mice. Analyses were performed 2 weeks after the initial tamoxifen administration. **(B,C)** Images and comparisons of CD13^+^-pericyte coverage over lectin^+^-vessels, and capillary density, capillary length, and capillary tortuosity in vehicle-treated control (*n* = 6) and DTA^*i*△PC^ (*n* = 8) mice. **(D)** Representative image of capillary in two-photon microscopy (TPM) imaging. White arrows indicate that the line scanning directions to measure capillary diameter and RBC velocity. **(E)** Paired comparisons of capillary diameter, RBC velocity, and RBC volume flux in control (*n* = 5) and DTA^*i*△PC^ (*n* = 4) mice. Imaging is performed twice, before tamoxifen administration (baseline) and 2 weeks after the initial tamoxifen administration (after 2 weeks), in the same mice. The measurements from at least 10 capillaries for each mouse were averaged. Error bars represent mean ± *SD*. ^∗∗∗∗^*P* < 0.0001 and ^∗^*P* < 0.05 vs. control, by Welch’s *t*-test for unpaired comparison analyses and Student’s *t*-test for paired comparison analyses. Scale bars: 100 μm (**B**, white) and 10 μm (**D**, yellow). PDGFRβ, platelet-derived growth factor receptor beta; DTA, diphtheria toxin; IHC, immunohistochemistry; WB, western blot; RBC, red blood cell.

PDGFRβ-CreER^T2^ mice and ROSA26-DTA mice were crossed to deplete pericytes by diphtheria toxin A (DTA) expression in a PDGFRβ-dependent tamoxifen-inducible manner (DTA^*i*△PC^) (Voehringer et al., 2008; Sheikh et al., 2015; Park et al., 2017). To deplete pericytes, 2 mg of tamoxifen dissolved in corn oil was injected intraperitoneally for five consecutive days (Park et al., 2017; Nikolakopoulou et al., 2019; Figure 1A). CD13^+^-pericyte coverage was significantly reduced in the brain vessels of DTA^*i*△PC^ mice (56.80% coverage vs. control mice), consistent with previous finding in retinal vessels (Park et al., 2017). No significant differences were found in capillary structure, such as capillary density, capillary length, and capillary tortuosity (Figures 1B,C). However, the capillary diameter was significantly enlarged in pericyte-deficient mice without a significant change in RBC velocity. In addition, the RBC volume in the capillaries showed no significant difference in DTA^*i*△PC^ mice compared to control mice (Figures 1D,E). These results indicate that pericyte loss has no effect on RBC flow in non-stalled capillaries. Next, we investigated BBB leakage, because pericyte is known to contribute to BBB integrity (Armulik et al., 2010; Daneman et al., 2010; Nikolakopoulou et al., 2019). However, there was no BBB leakage in pericyte-deficient mice when it was accessed in two ways: intravenous injection of cadaverine (950 Da), and IgG staining (200 kDa) (Supplementary Figure 2). These results indicate that partial loss of pericytes is not sufficient to interfere with BBB integrity during adulthood. Furthermore, glial fibrillary acidic protein (GFAP) expression, a marker for astrocyte activation, was significantly increased in pericyte-deficient mice (Supplementary Figure 3). This indicates that pericyte loss induces gliosis in the brain.

### Pericyte Loss Increases Capillary Stalling

**FIGURE 2:**
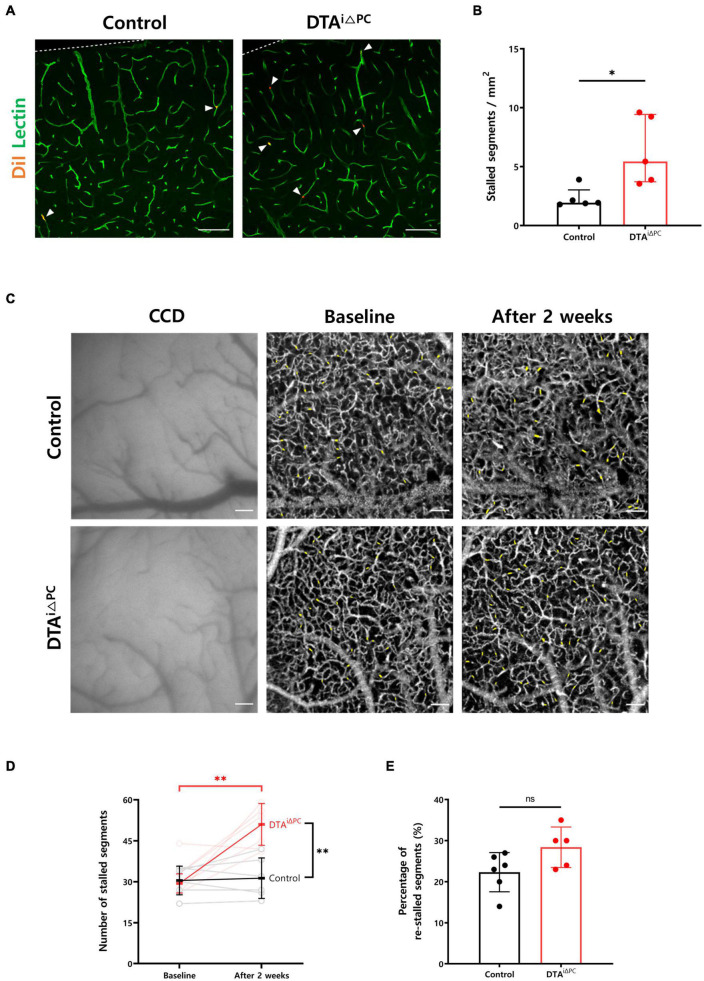
Capillary stalling is increased in pericyte-deficient mice. **(A,B)** Images and comparison of the number of capillary segments labeled with DiI-coated microspheres in control (*n* = 5) and DTA^*i*△PC^ (*n* = 5) mice. The number of capillary segments labeled with DiI-coated microspheres was measured in the primary somatosensory cortex. The measurements from at least 10 capillaries for each mouse were averaged. **(C)** CCD and time-averaged OCTA images at different time points showing no structural changes both in control and DTA^*i*△PC^ mice. Time-averaged OCTA images were obtained by averaging 10 angiograms acquired in 63 s. Imaging was performed twice, before tamoxifen administration (baseline) and 2 weeks after the initial tamoxifen administration (after 2 weeks), in the same mice. Positions of stalled segments are shaded in yellow. **(D,E)** Comparisons of the number of stalled segments and percentage of re-stalled segments in control (*n* = 6) and DTA^*i*△PC^ (*n* = 5) mice. The number of re-stalled segments after 2 weeks was normalized to the number of stalled segments at baseline. Error bars represent median with interquartile range and mean ± *SD*. ^∗^*P* < 0.05 vs. control, by Mann-Whitney test for comparison analysis of the number of capillary segments labeled with DiI-coated microspheres. ^∗∗^*P* < 0.01 vs. control, by Student’s *t*-test for paired comparison analysis of the number of stalled segments and Welch’s *t*-test for unpaired comparison analysis of the number of stalled segments. All scale bars are 100 μm. CCD, charge-coupled device; OCTA, optical coherence tomography angiography.

To investigate the effect of pericyte loss on capillary stalling, we compared the number of stalled segments in the capillaries between experimental groups. The number of capillary segments labeled with DiI-coated microspheres was nearly three times higher in pericyte-deficient mice (Figures 2A,B). Furthermore, we conducted *in vivo* label-free imaging of cerebral microcirculation using OCTA to measure the number of stalled segments (Figure 2C). Consistent with our finding in *ex vivo* analysis, the number of stalled segments was significantly increased in pericyte-deficient mice compared to control mice (Figure 2D). However, the percentage of re-stalled segments, which is the ratio of the capillary segments stalled again after 2 weeks, was not significantly different between the experimental groups (Figure 2E). Overall, these results indicate that pericyte loss increases capillary stalling in the brain.

### Pericyte Loss Decreases the Extent of the Cerebral Endothelial Glycocalyx

**FIGURE 3:**
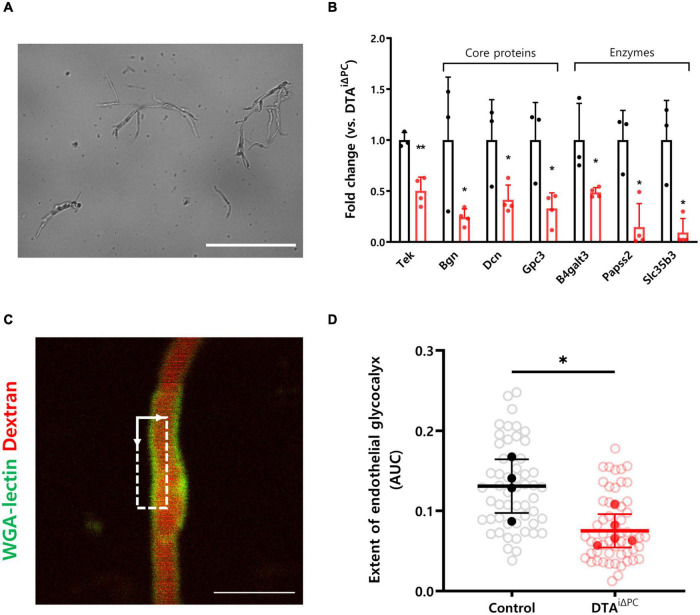
The extent of the cerebral endothelial glycocalyx is decreased in pericyte-deficient brain capillaries. **(A)** Representative image of isolated brain capillaries. **(B)** Comparisons of the transcription profiles of genes contributing to maintaining structure of glycocalyx. List of genes was selected based on previous studies and only those that showed significant difference (*P* < 0.05) were selected. Capillaries were isolated from the brain of control (*n* = 3) and DTA^*i*△PC^ (*n* = 4) mice. (C) TPM image of WGA lectin^+^-glycocalyx in capillary. Dotted square in TPM image indicates the region for analysis of the cerebral endothelial glycocalyx extent. (see supplementary Figure 3 for details.) **(D)** Comparison of the extent of the cerebral endothelial glycocalyx in control (54 capillaries, *n* = 4) and DTA^*i*△PC^ (56 capillaries, *n* = 5) mice. White circles represent the individual value of the cerebral endothelial glycocalyx extent for each capillary. Black and red circles represent the averaged value of the cerebral endothelial glycocalyx extent for each mouse. The averaged values of the cerebral endothelial glycocalyx extent for each mouse are utilized for comparison analysis. The measurements from at least 10 capillaries for each mouse were averaged. Error bars represent mean ± SD. ^∗∗^*P* < 0.01 and ^∗^*P* < 0.05 vs. control, by Student’s *t*-test for comparison analyses of gene transcription profiles. ^∗^*P* < 0.05 vs. control, by Welch’s *t*-test for comparison analysis of the cerebral endothelial glycocalyx extent. Scale bars: 100 μm **(A)** and 10 μm **(C)**. Tek, tek receptor tyrosine kinase; Bgn, biglycan; Dcn, decorin; Gpc3, glypican 3; B4galt3, beta-1,4-galactosyltransferase 3; Papss2, 3′-phosphoadenosine 5′-phosphosulfate synthase 2; Slc35b3, solute carrier family 35 member b3; TPM, two-photon microscopy; WGA-lectin, wheat germ agglutinin lectin; AUC, area under the curve.

To understand the molecular processes underlying increased capillary stalling, we performed RNA-Seq of brain capillaries after isolation from the brain (Figure 3A and Supplementary Figure 4). Interestingly, genes that are known to maintain the structure of the endothelial glycocalyx, such as Tek, Bgn, Dcn, Gpc3, B4galt3, Papss2, and Slc35b3, were significantly downregulated in the brain capillaries of pericyte-deficient mice (Figure 3B). In addition, a previous study reported that the extent of the cerebral endothelial glycocalyx is smaller in the stalled segments of the capillaries (Yoon et al., 2022). Therefore, we measured the extent of the cerebral endothelial glycocalyx in pericyte-deficient mice by using the region that best matched the capillary image plane (dotted square in Figure 3C). Our findings showed that the extent of the cerebral endothelial glycocalyx was significantly decreased in pericyte-deficient mice (Figure 3D). These results show that pericyte loss induces the decrease of the extent of the cerebral endothelial glycocalyx.

### Leukocyte Adhesion Molecules Are Highly Expressed in Pericyte-Deficient Mice

**FIGURE 4:**
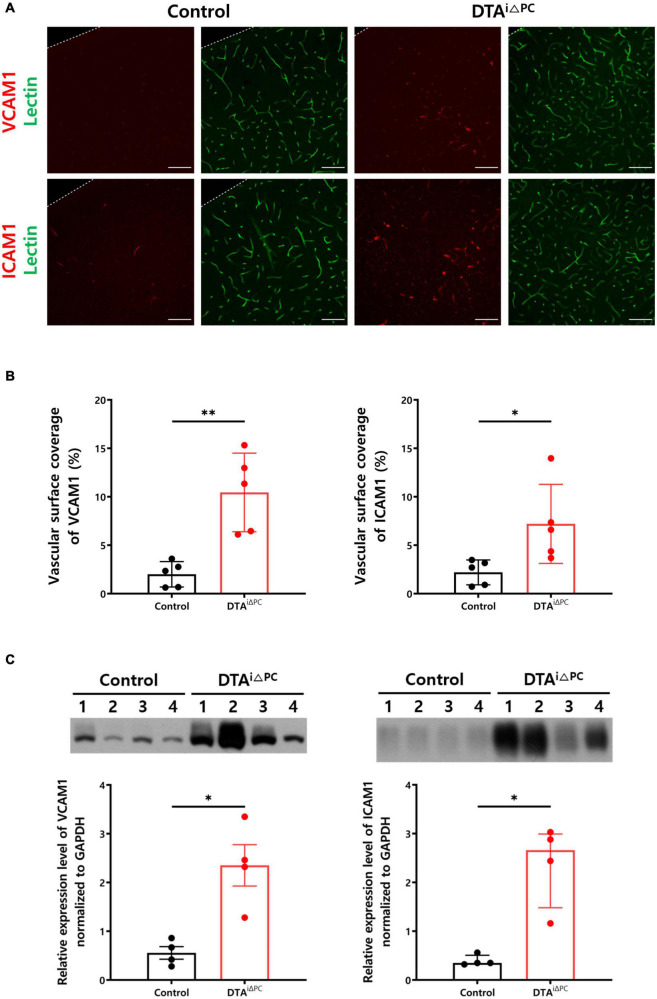
Pericyte loss leads to increased expression of leukocyte adhesion molecules. **(A,B)** Images and comparisons of VCAM1^+^ and ICAM1^+^ coverage over lectin^+^-vessels in control (*n* = 5) and DTA^*i*△PC^ (*n* = 5) mice. **(C)** Comparisons of VCAM1 and ICAM1 expression in the whole brain lysate in control (*n* = 4) and DTA^*i*△PC^ (*n* = 4) mice. GAPDH control is shown in Supplementary Figure 6A. Error bars represent mean ± SD and median with interquartile range. ^∗∗^*P* < 0.01 and ^∗^*P* < 0.05 vs. control, by Welch’s *t*-test for comparison analyses of vascular surface coverage of VCAM1, vascular surface coverage of ICAM, VCAM expression in the whole brain lysate. ^∗^*P* < 0.05 vs. control, by Mann-Whitney test for comparison analysis of ICAM1 expression in the whole brain lysate. All scale bars are 100 μm. VCAM1, vascular cell adhesion molecule 1; ICAM1, intercellular adhesion molecule 1; GAPDH, glyceraldehyde-3-phosphate dehydrogenase.

We found that the vascular surface coverage of VCAM1 and ICAM1 was significantly increased in pericyte-deficient brain vessels (Figures 4A,B). Consistently, VCAM1 and ICAM1 expression was significantly increased in the brain lysate of pericyte-deficient mice (Figure 4C). These results indicate that pericyte loss leads to increased expression of leukocyte adhesion molecules. In addition, to test whether the increase of VCAM1 and ICAM1 expression due to pericyte loss may be linked to the angiopoietin-Tie2 signaling pathway, we first examined Tie2 expression in vessels, because RNA-Seq results from the brain capillaries showed transcriptional inactivation of Tek (Tie2 coding gene) in pericyte-deficient mice (Figure 3B). As expected, Tie2 signal intensity in vessels was significantly decreased in pericyte-deficient mice (Supplementary Figures 5A,B). Furthermore, Tie2 expression was significantly decreased in the brain lysate of pericyte-deficient mice (Supplementary Figure 5C). Unexpectedly, however, there were no significant differences in p-Tie2, p-Tie2/Tie2, NFκB, p-NFκB, p-NFκB/NFκB, Angiopoietin1, Angiopoietin2, and Tie1 expression (Supplementary Figure 6). These results show that pericyte loss induces the decrease of Tie2 expression, however, it does not affect Angiopoietin1 and Angiopoietin2 expression, Tie2 and NFκB activity in the brain.

### Cerebral Hypoxia Is Induced by Pericyte Loss

**FIGURE 5:**
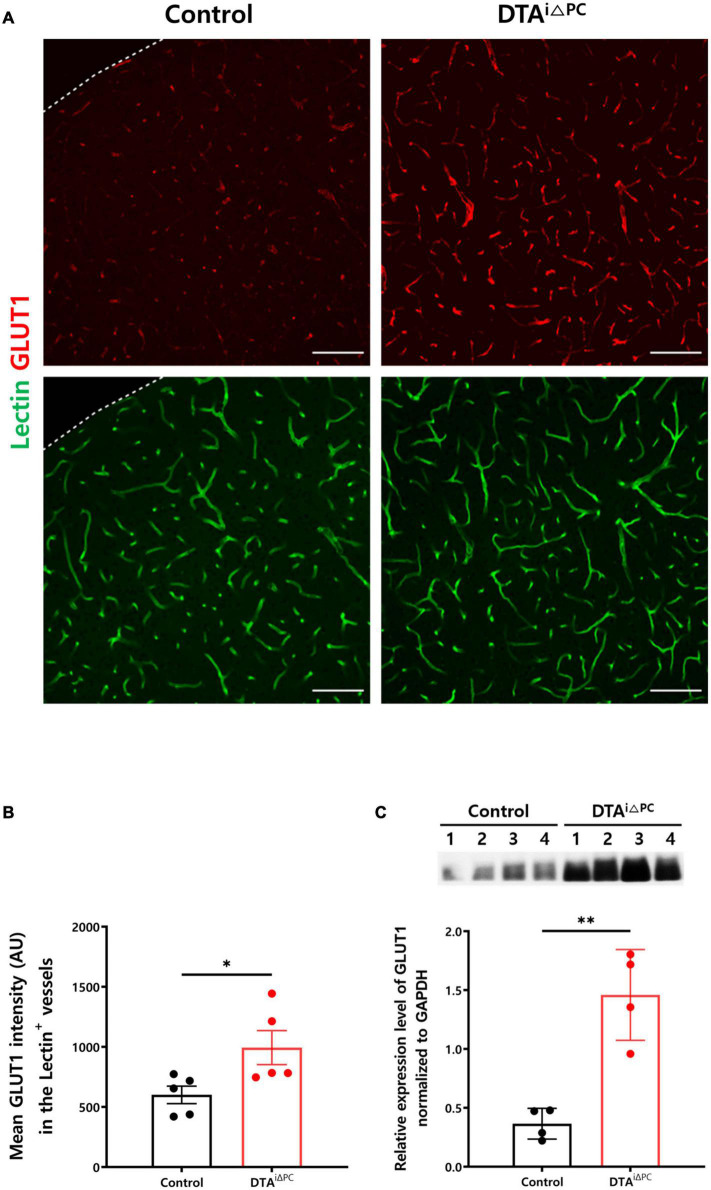
Cerebral hypoxia is induced in pericyte-deficient mice. **(A,B)** Images and comparisons of mean GLUT1 intensity in lectin^+^-vessels in control (*n* = 5) and DTA^*i*△PC^ (*n* = 5) mice. **(C)** Comparisons of GLUT1 expression in the whole brain lysate in control (*n* = 4) and DTA^*i*△PC^ (*n* = 4) mice. GAPDH control is shown in Supplementary Figure 6A. Error bars represent mean ± SD. ^∗^*P* < 0.05 and ^∗∗^*P* < 0.01 vs. control, by Welch’s *t*-test for comparison analyses. All scale bars are 100 μm. GLUT1, glucose transporter 1; GAPDH, glyceraldehyde-3-phosphate dehydrogenase.

To investigate the development of cerebral hypoxia, we measured glucose transporter 1 (GLUT1) expression in the vessels. GLUT1 signal intensity in the vessels was significantly increased in the pericyte-deficient mice (Figures 5A,B). Consistently, GLUT1 expression in the brain lysate was significantly increased in pericyte-deficient mice (Figure 5C). In addition, Hypoxyprobe-1 (pimonidazole HCl) signal intensity had a trend toward an increase (*p* = 0.0724) in pericyte-deficient mice (Supplementary Figure 7). These results indicate that pericyte loss induces cerebral hypoxia.

## Discussion

In this study, we demonstrated that pericyte loss induced capillary stalling in the brain without changing the capillary structure and BBB integrity. The capillary diameter was enlarged approximately 25% after pericyte loss, consistent with previous findings that showed that pericyte regulates basal capillary tone (Peppiatt et al., 2006; Berthiaume et al., 2018a). Although the capillary diameter was increased, the RBC velocity and RBC volume flux were not changed after pericyte loss. According to several previous studies, however, pericyte loss induces BBB leakage and CBF downregulation (Armulik et al., 2010; Daneman et al., 2010; Nikolakopoulou et al., 2019). This discrepancy might be due to differences in animal model. Furthermore, the RBC volume flux that was measured would be the value in non-stalled capillaries. Because the RBC volume flux in stalled capillaries would be negligible, the CBF which corresponds to the sum of the RBC volume flux would be decreased in pericyte-deficient mice. Our results suggest that capillary stalling is a mediating process in pericyte loss inducing CBF downregulation.

We measured capillary stalling in two ways: *ex vivo* analyses using DiI-coated microspheres and *in vivo* analyses using longitudinal OCTA imaging and found increased capillary stalling in pericyte-deficient mice in either of two ways. Moreover, the number of stalled segments in capillaries was higher in *in vivo* than in *ex vivo* analyses. Presumably, this discrepancy resulted from the difference in the temporal resolution of the two methods (3 h for the DiI-coated microspheres and only 6.3 s for longitudinal OCTA imaging). Indeed, the higher the temporal resolution the higher the sensitivity to detect capillary stalling is.

The increased interaction between leukocytes and leukocyte adhesion molecules fosters the adhesion of rolling leukocytes on the endothelial cell surface (Ley et al., 2007; Nourshargh and Alon, 2014). We measured the extent of the cerebral endothelial glycocalyx as a possible cause of increased capillary stalling because of its established association to leukocyte-endothelial cell interaction (Reitsma et al., 2007; Uchimido et al., 2019). Previous studies have shown that the endothelial glycocalyx is degraded in diseases, leading to increased leukocyte adhesion (Kumase et al., 2010; Schmidt et al., 2012; McDonald et al., 2016). Furthermore, the decreased extent of the cerebral endothelial glycocalyx is associated with increased capillary stalling (Yoon et al., 2022). RNA-seq results also suggested that leukocyte-endothelial cell interaction could be enhanced in pericyte-deficient mice. The adhesive interaction between leukocyte and endothelial cell is regulated by sequential activation of different families of membrane proteins (Panés and Granger, 1998). Taken together, it could be inferred that increased capillary stalling induced by pericyte loss is associated with increased leukocyte adhesion to endothelial cell due to the decreased extent of the cerebral endothelial glycocalyx and increased expression of leukocyte adhesion molecules. In addition, we investigated Tie2 signaling as the main upstream pathway of increased expression of leukocyte adhesion molecules. Previous studies have shown that the enhanced expression of VCAM1 and ICAM1 is induced by the activation and nuclear translocation of the inflammatory transcription factor NFκB, which is a downstream signaling molecule of the angiopoietin-Tie2 signaling pathway (Parikh, 2017). In addition, pericyte produces Angiopoietin1, an agonistic ligand of Tie2 (Augustin et al., 2009; Saharinen and Alitalo, 2011). We therefore hypothesized that the decrease of Angiopoietin1 due to pericyte loss may deactivate Tie2 signaling, and in turn, increase VCAM1 and ICAM1 expression. Although transcriptional inactivation of Tie2 coding gene and decreased expression of Tie2 were observed, there was no significant difference in the Tie2 signaling pathway. This result suggests that the angiopoietin-Tie2 signaling may not be involved in the increased expression of leukocyte adhesion molecules in the brain. Previous study reported that Tie2 is expressed at lower levels by pericytes, and pericyte-expressed Tie2 also controls angiogenesis and vessel maturation, which suggests that pericyte-expressed Tie2 contributes to maintaining the homeostasis of the vasculature (Teichert et al., 2017). It is known that the expression of leukocyte adhesion molecules is increased during inflammation (Ley et al., 2007). Furthermore, increasing evidence points to the role of pericyte to maintain immune homeostasis in the brain (Rustenhoven et al., 2017). Taken together, these results suggest that the cerebral inflammation induced by pericyte loss leads to increased expression of leukocyte adhesion molecules. The combination of the increased expression of leukocyte adhesion molecules and increased exposure of leukocyte adhesion molecules due to the decreased extent of the cerebral endothelial glycocalyx leads to increased interaction between leukocytes and leukocyte adhesion molecules, which results in increased capillary stalling. When the regulation of cerebral blood flow is not working properly, the oxygenated blood supply to the brain tissue is limited (Kisler et al., 2017), which in turn leads to hypoxia in the brain (Bell et al., 2010; Nikolakopoulou et al., 2019). Considering the profound effect of capillary stalling on CBF downregulation (Cruz Hernández et al., 2019), increased capillary stalling would limit the oxygenated blood supply to the brain tissue. In turn, cerebral hypoxia and augmented gliosis were induced in pericyte-deficient mice, which may lead to the vicious cycle of increasing capillary stalling.

This study has some limitations. First, OCTA and TPM imagings were performed only in the cortex of mice due to the relatively shallow imaging depth of the imaging modalities. In addition, arterial blood flow was not measured to show upstream blood flow change after pericyte loss. Since we used the whole brain lysate to investigate the angiopoietin-Tie2 signaling pathway in pericyte-deficient mice, it could provide a limited opportunity to detect the change in capillaries. Nevertheless, our results clearly demonstrated increased capillary stalling in the pericyte-deficient mice compared to the control.

## Conclusion

In conclusion, this study showed that pericyte loss resulted in increased capillary stalling in the brain through increased interaction between leukocyte and endothelial cell, which was mediated by the decreased extent of the cerebral endothelial glycocalyx and increased expression of leukocyte adhesion molecules. Furthermore, pericyte loss induced hypoxia and gliosis. This study provides clues regarding pericyte loss-inducing alterations in microcirculation, and we expect that pericyte could be a potential therapeutic target for microcirculatory dysfunction in neurological diseases.

## Data Availability Statement

The data presented in the study are deposited in Sequence Read Archive (SRA, https://www.ncbi.nlm.nih.gov/sra), Accession number: PRJNA806992.

## Ethics Statement

The animal care and experimental procedures in this study were performed with the approval of the Institutional Animal Care and Use Committee (Nos. KA2018-32 and KA2021-46) of KAIST and in accordance with the ARRIVE guidelines (Percie du Sert et al., 2020).

## Author Contributions

Y-GC, J-HY, and YJ designed the experiments, discussed the results, wrote, and edited the manuscript. Y-GC, J-HY, JJ, and BK performed the experiments. Y-GC, JJ, and BK analyzed the data. SH and GK provided the mice. SH, GK, D-SL, and W-YO provided critical comments on this study. W-YO and YJ supervised the project. All authors contributed to the article and approved the submitted version.

## Conflict of Interest

The authors declare that the research was conducted in the absence of any commercial or financial relationships that could be construed as a potential conflict of interest.

## Publisher’s Note

All claims expressed in this article are solely those of the authors and do not necessarily represent those of their affiliated organizations, or those of the publisher, the editors and the reviewers. Any product that may be evaluated in this article, or claim that may be made by its manufacturer, is not guaranteed or endorsed by the publisher.

## Footnotes

1
https://github.com/uva-peirce-cottler-lab/public_REAVER

2
http://www.gsea-msigdb.org/gsea/index.jsp

3
https://www.gsea-msigdb.org/gsea/msigdb/
