# Supplementary material for: Pericyte Loss Leads to Capillary Stalling Through Increased Leukocyte-Endothelial Cell Interaction in the Brain
Source: Front Cell Neurosci. 2022 Mar 11;16:848764. doi: 10.3389/fncel.2022.848764 (PMC8962364; doi:10.3389/fncel.2022.848764)
Supplement: Supplementary file 1 [file Data_Sheet_1.pdf]

Supplementary Material

This document includes:

1. Supplementary figure 1 to 7

2. Supplementary table 1

1 Supplementary figures

A

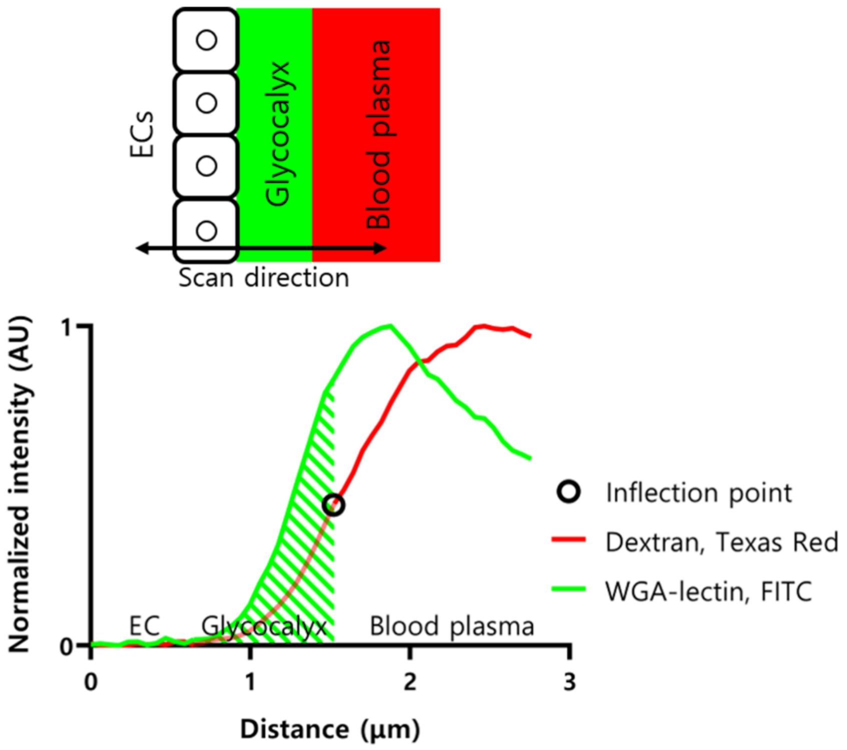

Supplementary figure 1. An illustration showing the cerebral endothelial glycocalyx layer and TPM scan direction

(A) The averaged intensity profile of WGA-lectin (green) and dextran (red) along the scan direction together with the inflection point of the dextran curve (circle) from the capillary in Figure 3C are shown. Each profile is minimum-maximum normalized for better representation.

EC, endothelial cell; TPM, two-photon microscopy; WGA-lectin, wheat germ agglutinin lectin; AU, arbitrary unit; AUC, area under the curve.

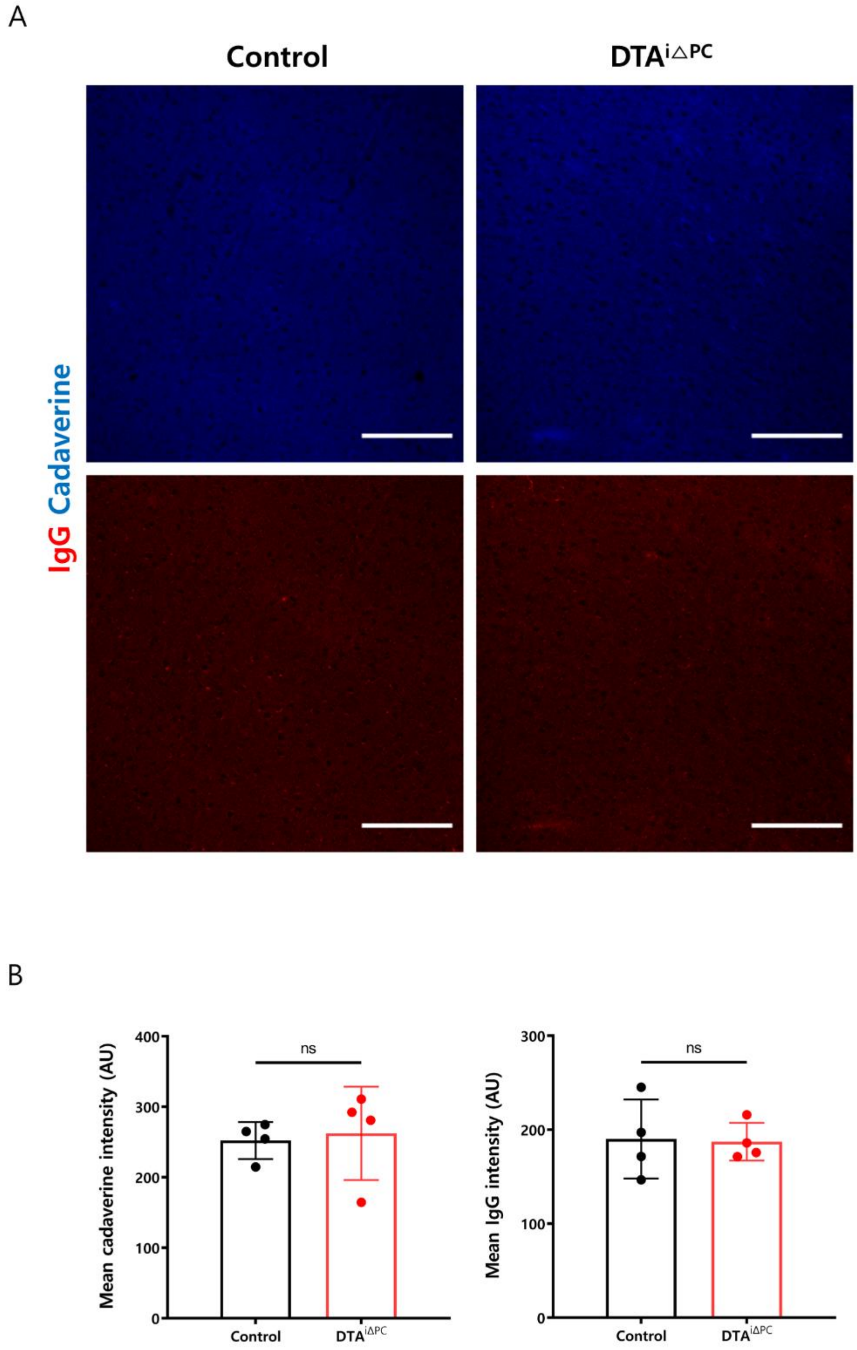

Supplementary figure 2. BBB leakage is not observed in pericyte-deficient mice

(A,B) Images and comparisons of cadaverine and IgG leakage in control (n = 4) and DTA$^{i\triangle PC}$ (n = 4) mice. Cadaverine was infused intravenously, 3 h before sacrifice.

Error bars represent mean±SD. Welch's t-test for comparison analyses. All scale bars are 100 μm. BBB, blood-brain barrier; IgG, immunoglobulin G; AU, arbitrary unit.

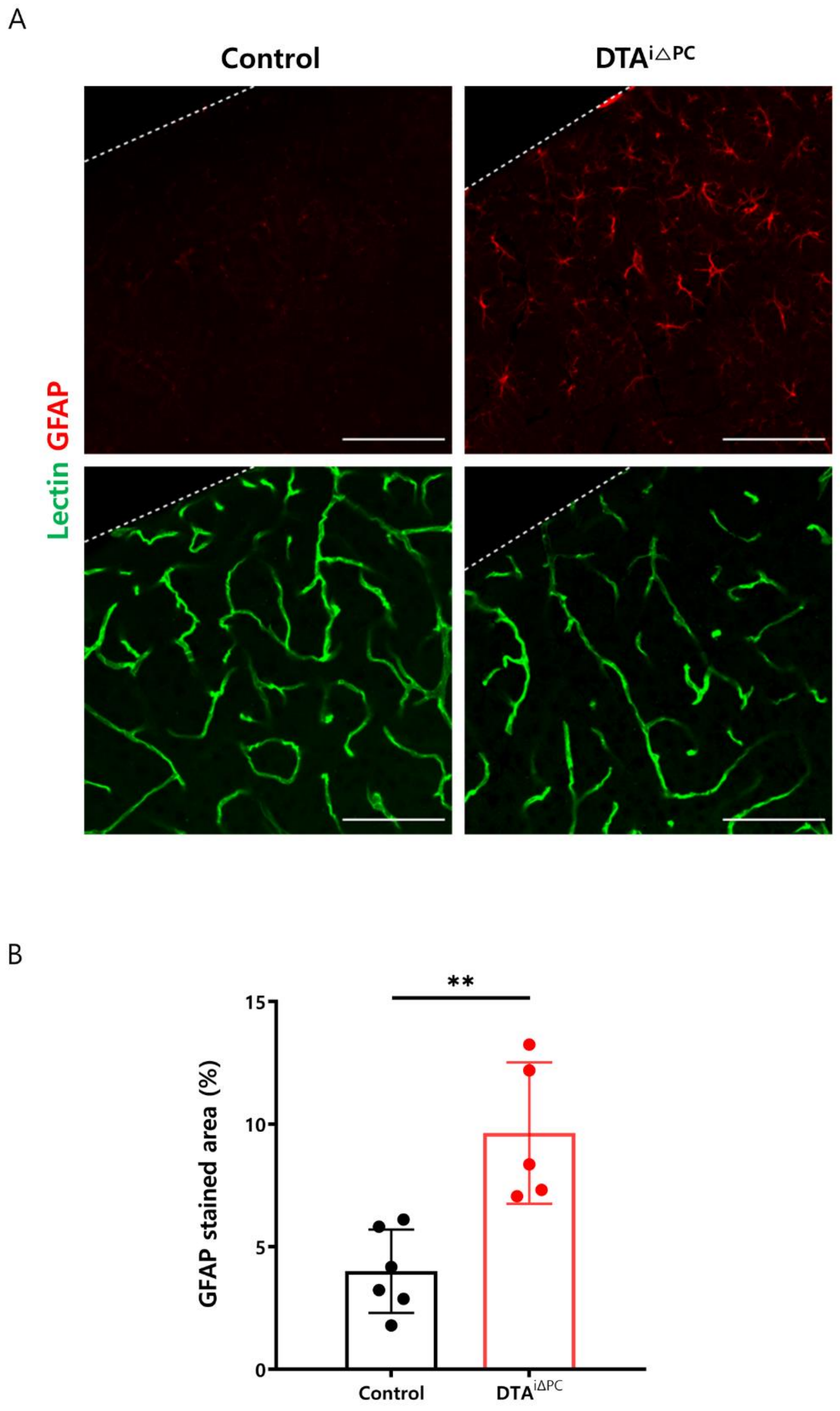

Supplementary figure 3. Gliosis is increased in pericyte-deficient mice

(A,B) Images and comparisons of GFAP expression in control (n = 6) and $DTA^{i\triangle PC}$ (n = 5) mice.

Error bars represent mean±SD. ***P*<0.01 versus control, by Welch's t-test for comparison analysis. All scale bars are 100 μm. GFAP, glial fibrillary acidic protein.

A

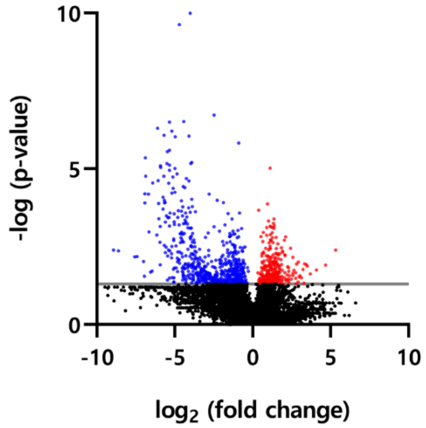

B

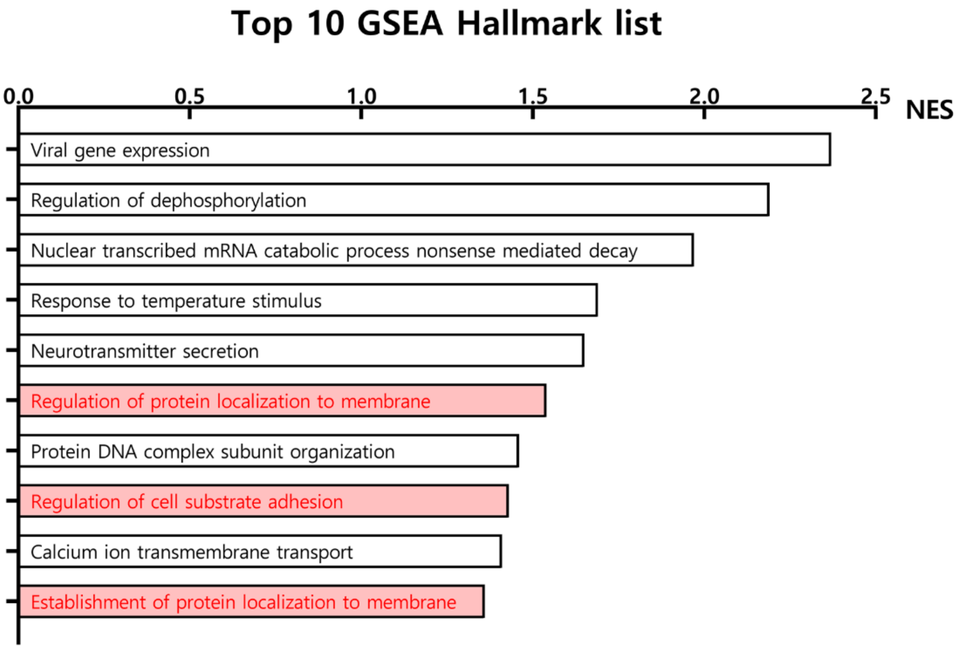

Supplementary figure 4. RNA-Seq results from the brain capillaries

(A) Volcano plot of differentially expressed genes (DEGs) in pericyte-deficient mice. The horizontal line represents the threshold for significance (P=0.05). Red dots indicate upregulated genes. Blue dots indicate downregulated genes. n = 3 samples for control and n = 4 samples for DTA$^{i\triangle PC}$ mice.

(B) Top ten enriched gene sets of the C5 category in pericyte-deficient mice. The three gene sets related to leukocyte-endothelial cell interaction are highlighted in red.

Student’s t-test for comparison analysis. GSEA, gene set enrichment analysis; NES, normalized enrichment score.

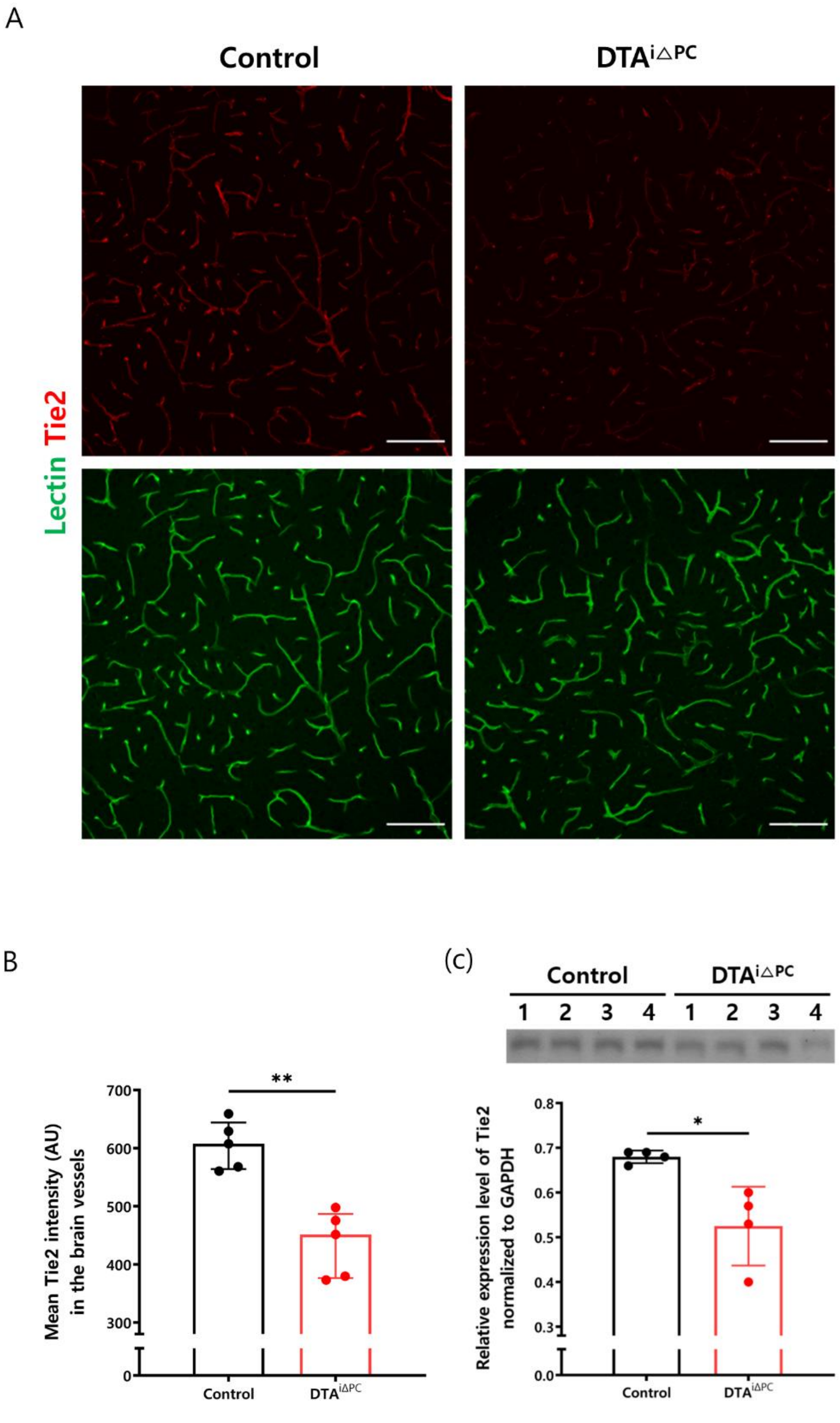

Supplementary figure 5. Tie2 expression is decreased in pericyte-deficient mice

(A,B) Images and comparisons of Tie2 expression in lectin^{+}-vessels in control (n = 5) and DTA$^{i\triangle PC}$ (n = 5) mice.

(C) Comparison of Tie2 expression in the whole brain lysate in control (n = 4) and $DTA^{i\triangle PC}$ (n = 4) mice. GAPDH control is shown in supplementary figure 6A.

Error bars represent median with interquartile range and mean±SD. $^{**}P<0.01$ versus control, by Mann-Whitney test for comparison analysis of mean Tie2 intensity in the vessels. $^{*}P<0.05$ versus control, by Welch’s t-test for comparison analysis of Tie2 expression in the whole brain lysate. All scale bars are 100 µm. AU, arbitrary unit; GAPDH, glyceraldehyde-3-phosphate dehydrogenase.

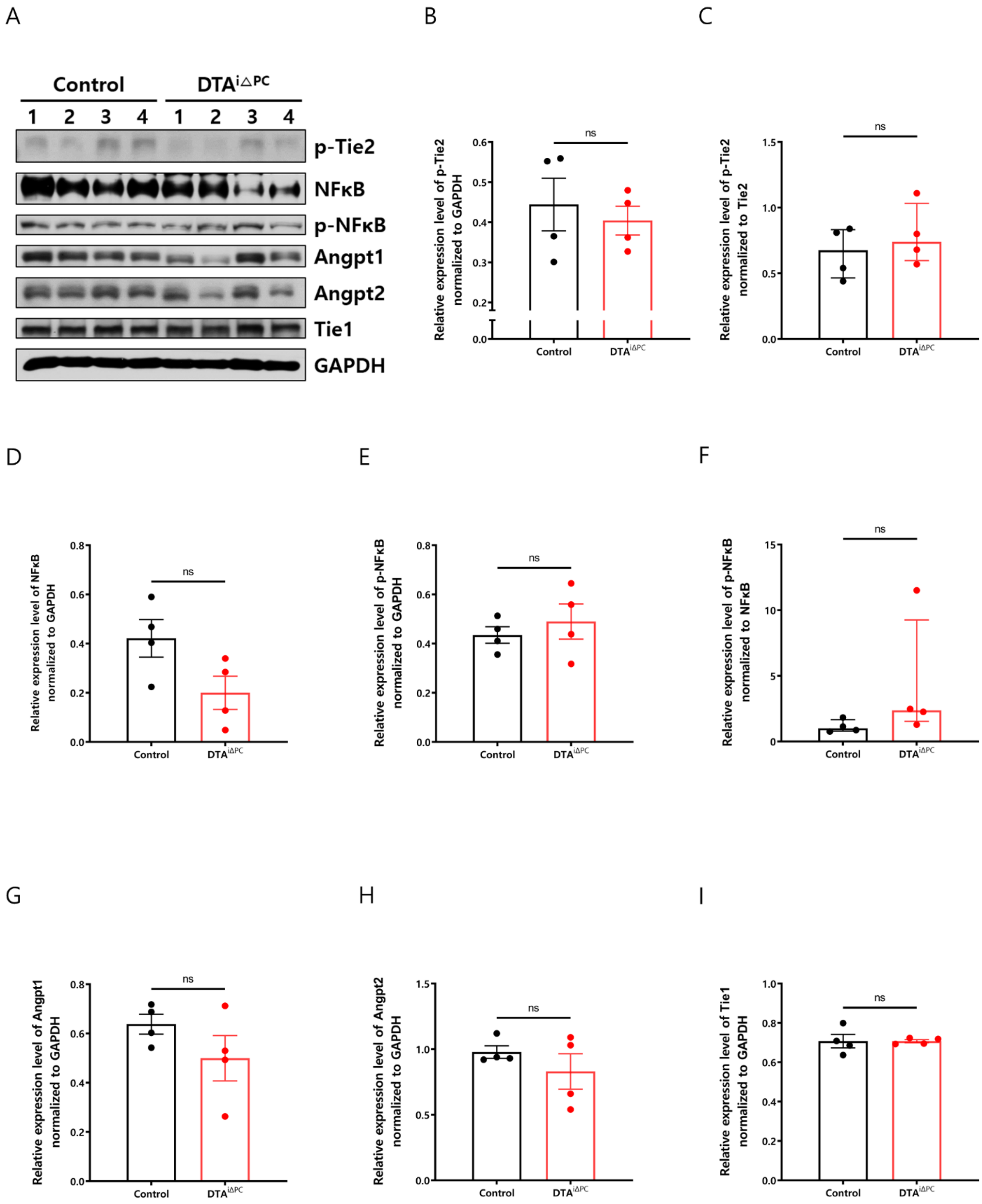
A
Control
DTAi△PC
1 2 3 4 1 2 3 4
p-Tie2
NFκB
p-NFκB
Angpt1
Angpt2
Tie1
GAPDH
B
Relative expression level of p-Tie2 normalized to GAPDH
ns
Control
DTAiΔPC
C
Relative expression level of p-Tie2 normalized to Tie2
ns
Control
DTAiΔPC
D
Relative expression level of NFκB normalized to GAPDH
ns
Control
DTAiΔPC
E
Relative expression level of p-NFκB normalized to GAPDH
ns
Control
DTAiΔPC
F
Relative expression level of p-NFκB normalized to NFκB
ns
Control
DTAiΔPC
G
Relative expression level of Angpt1 normalized to GAPDH
ns
Control
DTAiΔPC
H
Relative expression level of Angpt2 normalized to GAPDH
ns
Control
DTAiΔPC
I
Relative expression level of Tie1 normalized to GAPDH
ns
Control
DTAiΔPC

Supplementary figure 6. Angiopoietin-Tie2 signaling is unaffected in pericyte-deficient mice

(A) Western blot of p-Tie2, NFκB, p-NFκB, Angpt1, Angpt2, Tie1, and GAPDH in the whole brain lysate of control (n = 4) and $DTA^{i\triangle PC}$ (n = 4) mice.

Comparison of (B) p-Tie2, (C) p-Tie2/Tie2, (D) NFκB, (E) p-NFκB, (F) p-NFκB/NFκB, (G) Angpt1, (H) Angpt2, and (I) Tie1 expression.

Error bars represent mean±SD and median with interquartile range. Welch's t-test for comparison analyses of p-Tie2, p-Tie2/Tie2, NFκB, p-NFκB, Angpt1, Tie1 expression in the whole brain lysate. Mann-Whitney test was used for comparison analyses of p-NFκB/NFκB, Angpt2 expression in the whole brain lysate. NFκB, nuclear factor kappa B; Angpt1, Angiopoietin1; Angpt2, Angiopoietin2; GAPDH, glyceraldehyde-3-phosphate dehydrogenase.

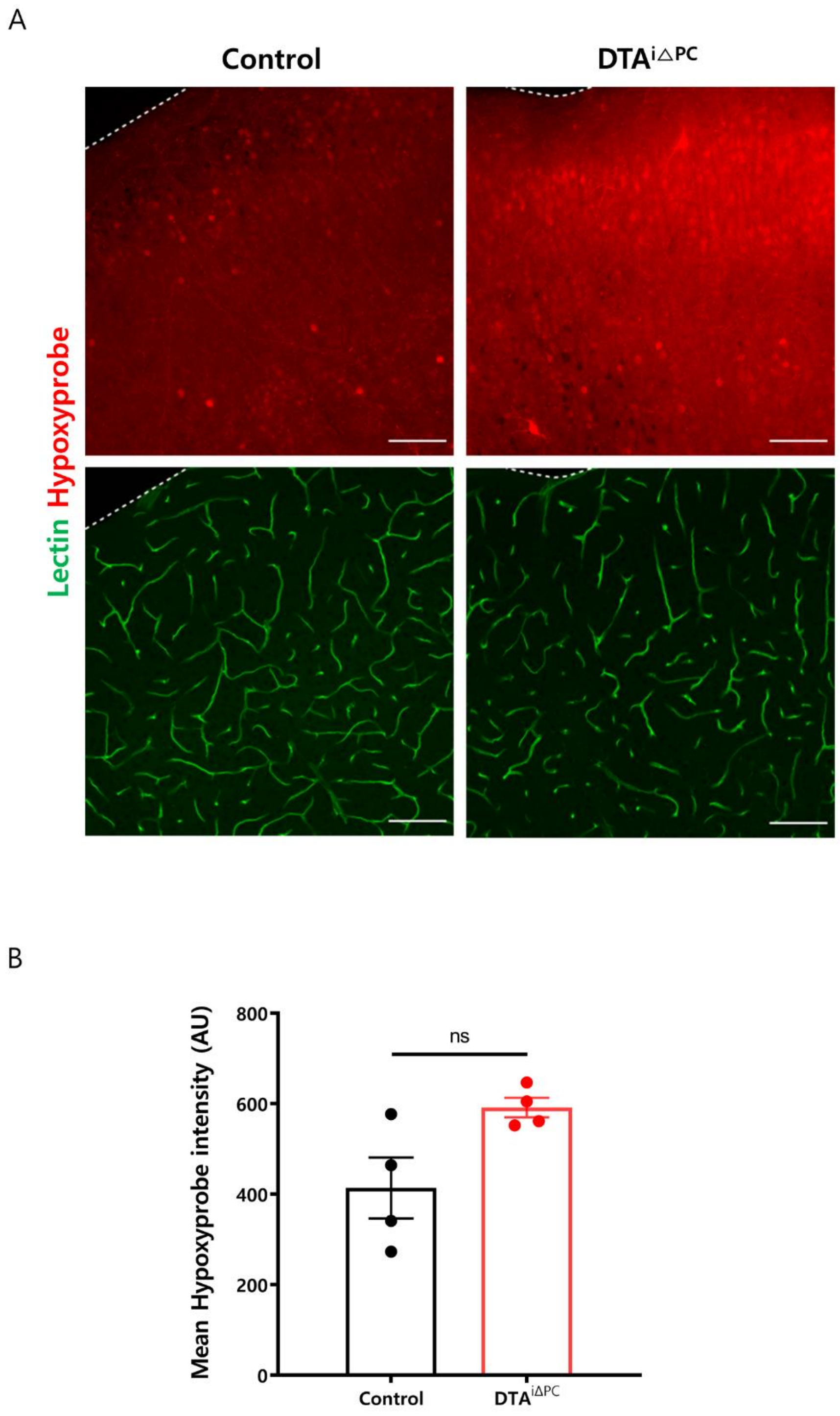

Supplementary figure 7. Hypoxyprobe intensity is increased in pericyte-deficient mice

(A,B) Images and comparisons of Hypoxyprobe-1 intensity in control (n = 4) and DTA$^{i\triangle PC}$ (n = 4) mice. Hypoxyprobe-1 was injected intraperitoneally, 1 h before sacrifice.

Error bars represent mean±SD. Welch's t-test for comparison analysis. All scale bars are 100 μm.

2 Supplementary table

Supplementary table 1. Statistical reporting

**Figure**	**N**<br>**(mouse number)**	**Data structure**<br>**(normality test)**	**P value**<br>**in normality test**	**Average**<br>**and variability**	**P value**<br>**(Test used)**
Figure 1C<br>Pericyte coverage	Control:<br>n = 6<br>$DTA^{i\triangle PC}$:<br>n = 8	Normal distribution<br>(Shapiro-Wilk test)	Control:<br>$P = 0.2014$<br>$DTA^{i\triangle PC}$:<br>$P = 0.5283$	Control:<br>56.38 ± 3.25<br>$DTA^{i\triangle PC}$:<br>32.02 ± 9.47	$P < 0.0001$<br>(Welch's t-test)
Figure 1C<br>Capillary density	Control:<br>n = 6<br>$DTA^{i\triangle PC}$:<br>n = 8	Normal distribution<br>(Shapiro-Wilk test)	Control:<br>$P = 0.4711$<br>$DTA^{i\triangle PC}$:<br>$P = 0.4283$	Control:<br>6.41 ± 0.33<br>$DTA^{i\triangle PC}$:<br>6.85 ± 0.85	$P = 0.2076$<br>(Welch's t-test)
Figure 1C<br>Capillary length	Control:<br>n = 6<br>$DTA^{i\triangle PC}$:<br>n = 8	Normal distribution<br>(Shapiro-Wilk test)	Control:<br>$P = 0.6601$<br>$DTA^{i\triangle PC}$:<br>$P = 0.9082$	Control:<br>17.85 ± 1.57<br>$DTA^{i\triangle PC}$:<br>18.02 ± 1.96	$P = 0.8529$<br>(Welch's t-test)

Figure 1C<br><br>Mean capillary tortuosity	Control:<br><br>n = 6<br><br>DTA$^{i\triangle PC}$:<br><br>n = 8	Normal distribution<br><br>(Shapiro-Wilk test)	Control:<br><br>*P* = 0.2782<br><br>DTA$^{i\triangle PC}$:<br><br>*P* = 0.2289	Control:<br><br>1.96 ± 0.02<br><br>DTA$^{i\triangle PC}$:<br><br>1.99 ± 0.07	*P* = 0.2696<br><br>(Welch's t-test)
Figure 1C<br><br>Averaged capillary diameter	Control:<br><br>n = 5<br><br>DTA$^{i\triangle PC}$:<br><br>n = 4	Normal distribution<br><br>(Shapiro-Wilk test)	Control (Baseline):<br><br>*P* = 0.2413<br><br>Control (After 2 weeks):<br><br>*P* = 0.7023<br><br>DTA$^{i\triangle PC}$ (Baseline):<br><br>*P* = 0.4427<br><br>DTA$^{i\triangle PC}$ (After 2 weeks):<br><br>*P* = 0.4422	Control (Baseline):<br><br>4.30 ± 0.21<br><br>Control (After 2 weeks):<br><br>4.36 ± 0.27<br><br>DTA$^{i\triangle PC}$ (Baseline):<br><br>4.07 ± 0.25<br><br>DTA$^{i\triangle PC}$ (After 2 weeks):<br><br>5.10 ± 0.48	*P* = 0.7133<br><br>(Paired student's t-test, Baseline vs After 2 weeks in Control)<br><br>*P* = 0.0328<br><br>(Paired student's t-test, Baseline vs After 2 weeks in DTA$^{i\triangle PC}$)<br><br>*P* = 0.2013<br><br>(Welch's t-test, Control vs DTA$^{i\triangle PC}$ at baseline)<br><br>*P* = 0.0453<br><br>(Welch's t-test, Control vs DTA$^{i\triangle PC}$ after 2 weeks)
Figure 1E<br><br>Averaged RBC velocity	Control:<br><br>n = 5	Normal distribution<br><br>(Shapiro-Wilk test)	Control (Baseline):<br><br>*P* = 0.4530	Control (Baseline):<br><br>1.12 ± 0.30	*P* = 0.8286

	DTA$^{i\triangle PC}$:<br><br>n = 4		Control (After 2 weeks):<br><br>*P* = 0.3680<br><br>DTA$^{i\triangle PC}$ (Baseline):<br><br>*P* = 0.6762<br><br>DTA$^{i\triangle PC}$ (After 2 weeks):<br><br>*P* = 0.6216	Control (After 2 weeks):<br><br>1.21 ± 0.93<br><br>DTA$^{i\triangle PC}$ (Baseline):<br><br>0.72 ± 0.28<br><br>DTA$^{i\triangle PC}$ (After 2 weeks):<br><br>0.88 ± 0.46	(Paired student's t-test, Baseline vs After 2 weeks in Control)<br><br>*P* = 0.6552<br><br>(Paired student's t-test, Baseline vs After 2 weeks in DTA$^{i\triangle PC}$)<br><br>*P* = 0.0821<br><br>(Welch's t-test, Control vs DTA$^{i\triangle PC}$ at baseline)<br><br>*P* = 0.5134<br><br>(Welch's t-test, Control vs DTA$^{i\triangle PC}$ after 2 weeks)
Figure 1E<br><br>Averaged RBC volume flux	Control:<br><br>n = 5<br><br>DTA$^{i\triangle PC}$:<br><br>n = 4	Normal distribution<br><br>(Shapiro-Wilk test)	Control (Baseline):<br><br>*P* = 0.7706<br><br>Control (After 2 weeks):<br><br>*P* = 0.6941<br><br>DTA$^{i\triangle PC}$ (Baseline):<br><br>*P* = 0.0711	Control (Baseline):<br><br>7.71 ± 2.86<br><br>Control (After 2 weeks):<br><br>7.39 ± 2.72<br><br>DTA$^{i\triangle PC}$ (Baseline):<br><br>6.43 ± 1.71	*P* = 0.8483<br><br>(Paired student's t-test, Baseline vs After 2 weeks in Control)<br><br>*P* = 0.3544<br><br>(Paired student's t-test, Baseline vs After 2 weeks in DTA$^{i\triangle PC}$)<br><br>*P* = 0.4332

			DTA$^{i\triangle PC}$ (After 2 weeks):<br><br>*P* = 0.4285	DTA$^{i\triangle PC}$ (After 2 weeks):<br><br>9.76 ± 6.66	(Welch's t-test, Control vs DTA$^{i\triangle PC}$ at baseline)<br><br>*P* = 0.5428<br><br>(Welch's t-test, Control vs DTA$^{i\triangle PC}$ after 2 weeks)
Figure 2B<br><br>Number of stalled segments	Control:<br><br>n = 5<br><br>DTA$^{i\triangle PC}$:<br><br>n = 5	Non-normal distribution<br><br>(Shapiro-Wilk test)	Control:<br><br>*P* = 0.0056<br><br>DTA$^{i\triangle PC}$:<br><br>*P* = 0.1593	Control:<br><br>1.94 (1.88-2.58)<br><br>DTA$^{i\triangle PC}$:<br><br>5.44 (3.89-6.39)	*P* = 0.0317<br><br>(Mann-Whitney test)
Figure 2D<br><br>Number of stalled segments	Control:<br><br>n = 6<br><br>DTA$^{i\triangle PC}$:<br><br>n = 5	Normal distribution<br><br>(Shapiro-Wilk test)	Control (Baseline):<br><br>*P* = 0.2392<br><br>Control (After 2 weeks):<br><br>*P* = 0.6022<br><br>DTA$^{i\triangle PC}$ (Baseline):<br><br>*P* = 0.1117<br><br>DTA$^{i\triangle PC}$ (After 2 weeks):<br><br>*P* = 0.4873	Control (Baseline):<br><br>29.60 ± 2.86<br><br>Control (After 2 weeks):<br><br>31.20 ± 8.29<br><br>DTA$^{i\triangle PC}$ (Baseline):<br><br>29.40 ± 3.51<br><br>DTA$^{i\triangle PC}$ (After 2 weeks):<br><br>51.00 ± 7.65	*P* = 0.6590<br><br>(Paired student's t-test, Baseline vs After 2 weeks in Control)<br><br>*P* = 0.0026<br><br>(Paired student's t-test, Baseline vs After 2 weeks in DTA$^{i\triangle PC}$)<br><br>*P* = 0.6886<br><br>(Welch's t-test, Control vs DTA$^{i\triangle PC}$ at baseline)

					P = 0.0022<br>(Welch's t-test, Control vs $DTA^{i\triangle PC}$ after 2 weeks)
Figure 2D<br>Percentage of re-stalled segments	Control:<br>n = 6<br>$DTA^{i\triangle PC}$:<br>n = 5	Normal distribution<br>(Shapiro-Wilk test)	Control:<br>*P* = 0.4084<br>$DTA^{i\triangle PC}$:<br>*P* = 0.4899	Control:<br>22.33 ± 4.76<br>$DTA^{i\triangle PC}$:<br>28.40 ± 4.93	*P* = 0.0708<br>(Welch's t-test)
Figure 3D<br>Extent of cerebral endothelial glycocalyx	Control:<br>n = 4<br>$DTA^{i\triangle PC}$:<br>n = 5	Normal distribution<br>(Shapiro-Wilk test)	Control:<br>*P* = 0.9710<br>$DTA^{i\triangle PC}$:<br>*P* = 0.7668	Control:<br>28.80 ± 4.09<br>$DTA^{i\triangle PC}$:<br>21.66 ± 2.64	*P* = 0.0353<br>(Welch's t-test)
Figure 4B<br>Vascular surface coverage of VCAM1	Control:<br>n = 5<br>$DTA^{i\triangle PC}$:<br>n = 5	Normal distribution<br>(Shapiro-Wilk test)	Control:<br>*P* = 0.3614<br>$DTA^{i\triangle PC}$:<br>*P* = 0.4197	Control:<br>2.01 ± 1.31<br>$DTA^{i\triangle PC}$:<br>10.45 ± 4.05	*P* = 0.0074<br>(Welch's t-test)
Figure 4B	Control:<br>n = 5	Normal distribution<br>(Shapiro-Wilk test)	Control:<br>*P* = 0.1823	Control:<br>2.20 ± 1.29	*P* = 0.0494<br>(Welch's t-test)

Vascular surface coverage of ICAM1	DTA$^{i\triangle PC}$:<br>n = 5		DTA$^{i\triangle PC}$:<br>*P* = 0.2193	DTA$^{i\triangle PC}$:<br>7.20 ± 4.08	
Figure 4C<br>Relative expression of VCAM1	Control:<br>n = 4<br>DTA$^{i\triangle PC}$:<br>n = 4	Normal distribution<br>(Shapiro-Wilk test)	Control:<br>*P* = 0.8322<br>DTA$^{i\triangle PC}$:<br>*P* = 0.8345	Control:<br>0.56 ± 0.26<br>DTA$^{i\triangle PC}$:<br>2.35 ± 0.85	*P* = 0.0196<br>(Welch's t-test)
Figure 4C<br>Relative expression of ICAM1	Control:<br>n = 4<br>DTA$^{i\triangle PC}$:<br>n = 4	Non-normal distribution<br>(Shapiro-Wilk test)	Control:<br>*P* = 0.0292<br>DTA$^{i\triangle PC}$:<br>*P* = 0.2343	Control:<br>0.35 (0.34-0.40)<br>DTA$^{i\triangle PC}$:<br>2.66 (2.12-2.92)	*P* = 0.0286<br>(Mann-Whitney test)
Figure 5B<br>Mean GLUT1 intensity in the vessels	Control:<br>n = 5<br>DTA$^{i\triangle PC}$:<br>n = 5	Normal distribution<br>(Shapiro-Wilk test)	Control:<br>*P* = 0.2485<br>DTA$^{i\triangle PC}$:<br>*P* = 0.0860	Control:<br>601.16 ± 162.54<br>DTA$^{i\triangle PC}$:<br>994.37 ± 315.92	*P* = 0.0483<br>(Welch's t-test)
Figure 5C<br>Relative expression of GLUT1	Control:<br>n = 4<br>DTA$^{i\triangle PC}$:	Normal distribution<br>(Shapiro-Wilk test)	Control:<br>*P* = 0.2071<br>DTA$^{i\triangle PC}$:	Control:<br>0.37 ± 0.13<br>DTA$^{i\triangle PC}$:	*P* = 0.0073<br>(Welch's t-test)

	n = 4		*P* = 0.5343	1.46 ± 0.39	
S. figure 2B<br><br>Mean cadaverine intensity	Control:<br><br>n = 4<br><br>DTA$^{i\triangle PC}$:<br><br>n = 4	Normal distribution<br><br>(Shapiro-Wilk test)	Control:<br><br>*P* = 0.3790<br><br>DTA$^{i\triangle PC}$:<br><br>*P* = 0.0988	Control:<br><br>252.23 ± 26.31<br><br>DTA$^{i\triangle PC}$:<br><br>262.24 ± 66.23	*P* = 0.7929<br><br>(Welch's t-test)
S. figure 2B<br><br>Mean IgG intensity	Control:<br><br>n = 4<br><br>DTA$^{i\triangle PC}$:<br><br>n = 4	Normal distribution<br><br>(Shapiro-Wilk test)	Control:<br><br>*P* = 0.8689<br><br>DTA$^{i\triangle PC}$:<br><br>*P* = 0.2779	Control:<br><br>190.23 ± 42.09<br><br>DTA$^{i\triangle PC}$:<br><br>187.27 ± 20.03	*P* = 0.9049<br><br>(Welch's t-test)
S. figure 3B<br><br>GFAP stained area	Control:<br><br>n = 6<br><br>DTA$^{i\triangle PC}$:<br><br>n = 5	Normal distribution<br><br>(Shapiro-Wilk test)	Control:<br><br>*P* = 0.6219<br><br>DTA$^{i\triangle PC}$:<br><br>*P* = 0.1712	Control:<br><br>4.00 ± 1.70<br><br>DTA$^{i\triangle PC}$:<br><br>9.64 ± 2.88	*P* = 0.0079<br><br>(Welch's t-test)
S. figure 5B<br><br>Mean Tie2 intensity in the vessels	Control:<br><br>n = 5<br><br>DTA$^{i\triangle PC}$:<br><br>n = 5	Non-normal distribution<br><br>(Shapiro-Wilk test)	Control:<br><br>*P* = 0.6533<br><br>DTA$^{i\triangle PC}$:<br><br>*P* = 0.3181	Control:<br><br>604.97 ± 41.41<br><br>DTA$^{i\triangle PC}$:<br><br>435.62 ± 56.45	*P* = 0.0079<br><br>(Mann-Whitney test)

S. figure 5C<br><br>Relative expression of Tie2	Control:<br><br>n = 4<br><br>$DTA^{i\triangle PC}$:<br><br>n = 4	Normal distribution<br><br>(Shapiro-Wilk test)	Control:<br><br>*P* = 0.1612<br><br>$DTA^{i\triangle PC}$:<br><br>*P* = 0.3890	Control:<br><br>0.68 ± 0.01<br><br>$DTA^{i\triangle PC}$:<br><br>0.53 ± 0.09	*P* = 0.0372<br><br>(Welch's t-test)
S. figure 6B<br><br>Relative expression of p-Tie2	Control:<br><br>n = 4<br><br>$DTA^{i\triangle PC}$:<br><br>n = 4	Normal distribution<br><br>(Shapiro-Wilk test)	Control:<br><br>*P* = 0.2001<br><br>$DTA^{i\triangle PC}$:<br><br>*P* = 0.5722	Control:<br><br>0.44 ± 0.13<br><br>$DTA^{i\triangle PC}$:<br><br>0.40 ± 0.07	*P* = 0.6158<br><br>(Welch's t-test)
S. figure 6C<br><br>Relative expression of p-Tie2/Tie2	Control:<br><br>n = 4<br><br>$DTA^{i\triangle PC}$:<br><br>n = 4	Normal distribution<br><br>(Shapiro-Wilk test)	Control:<br><br>*P* = 0.2945<br><br>$DTA^{i\triangle PC}$:<br><br>*P* = 0.6441	Control:<br><br>0.66 ± 0.20<br><br>$DTA^{i\triangle PC}$:<br><br>0.79 ± 0.23	*P* = 0.4204<br><br>(Welch's t-test)
S. figure 6D<br><br>Relative expression of NFκB	Control:<br><br>n = 4<br><br>$DTA^{i\triangle PC}$:<br><br>n = 4	Normal distribution<br><br>(Shapiro-Wilk test)	Control:<br><br>*P* = 0.9266<br><br>$DTA^{i\triangle PC}$:<br><br>*P* = 0.6132	Control:<br><br>0.42 ± 0.15<br><br>$DTA^{i\triangle PC}$:<br><br>0.20 ± 0.14	*P* = 0.0736<br><br>(Welch's t-test)
S. figure 6E	Control:	Normal distribution	Control:	Control:	*P* = 0.5217

Relative expression of p-NFκB	n = 4<br>$DTA^{i\triangle PC}$:<br>n = 4	(Shapiro-Wilk test)	*P* = 0.9855<br>$DTA^{i\triangle PC}$:<br>*P* = 0.9133	0.43 ± 0.07<br>$DTA^{i\triangle PC}$:<br>0.49 ± 0.14	(Welch's t-test)
S. figure 6F<br>Relative expression of p-NFκB/NFκB	Control:<br>n = 4<br>$DTA^{i\triangle PC}$:<br>n = 4	Non-normal distribution<br>(Shapiro-Wilk test)	Control:<br>*P* = 0.3300<br>$DTA^{i\triangle PC}$:<br>*P* = 0.0235	Control:<br>1.00 (0.84-1.31)<br>$DTA^{i\triangle PC}$:<br>2.37 (2.02-4.74)	*P* = 0.0571<br>(Mann-Whitney test)
S. figure 6G<br>Relative expression of Angpt1	Control:<br>n = 4<br>$DTA^{i\triangle PC}$:<br>n = 4	Normal distribution<br>(Shapiro-Wilk test)	Control:<br>*P* = 0.6799<br>$DTA^{i\triangle PC}$:<br>*P* = 0.8444	Control:<br>0.64 ± 0.08<br>$DTA^{i\triangle PC}$:<br>0.50 ± 0.18	*P* = 0.8587<br>(Welch's t-test)
S. figure 6H<br>Relative expression of Angpt2	Control:<br>n = 4<br>$DTA^{i\triangle PC}$:<br>n = 4	Non-normal distribution<br>(Shapiro-Wilk test)	Control:<br>*P* = 0.0149<br>$DTA^{i\triangle PC}$:<br>*P* = 0.3209	Control:<br>0.94 (0.93-0.99)<br>$DTA^{i\triangle PC}$:<br>0.85 (0.63-1.05)	*P* = 0.6857<br>(Mann-Whitney test)
S. figure 6I	Control:<br>n = 4	Normal distribution<br>(Shapiro-Wilk test)	Control:<br>*P* = 0.7808	Control:<br>0.71 ± 0.07	*P* = 0.9973<br>(Welch's t-test)

Relative expression of Tie1	DTA$^{i\triangle PC}$:<br>n = 4		DTA$^{i\triangle PC}$:<br>*P* = 0.1815	DTA$^{i\triangle PC}$:<br>0.71 ± 0.02	
Figure 7B<br>Mean Hypoxyprobe-1 intensity	Control:<br>n = 4<br>DTA$^{i\triangle PC}$:<br>n = 4	Normal distribution<br>(Shapiro-Wilk test)	Control:<br>*P* = 0.8358<br>DTA$^{i\triangle PC}$:<br>*P* = 0.5336	Control:<br>413.87 ± 134.55<br>DTA$^{i\triangle PC}$:<br>591.54 ± 43.50	*P* = 0.0724<br>(Welch’s t-test)
